# ‘It will harm business and increase illicit trade’: an evaluation of the relevance, quality and transparency of evidence submitted by transnational tobacco companies to the UK consultation on standardised packaging 2012

**DOI:** 10.1136/tobaccocontrol-2014-051930

**Published:** 2014-12-03

**Authors:** K A Evans-Reeves, J L Hatchard, A B Gilmore

**Affiliations:** Tobacco Control Research Group and member of UK Centre for Tobacco and Alcohol Studies, University of Bath, Bath, UK

**Keywords:** Tobacco industry, Public policy, Packaging and Labelling, Tobacco industry documents

## Abstract

**Introduction:**

Transnational tobacco companies (TTCs) submitted evidence to the 2012 UK Consultation on standardised packaging (SP) to argue the policy will have detrimental economic impacts and increase illicit tobacco trade.

**Methods:**

A content analysis of the four TTC submissions to the consultation assessed the relevance and quality of evidence TTCs cited to support their arguments. Investigative research was used to determine whether the cited evidence was industry connected. Fisher's exact tests were used to compare the relevance and quality of industry-connected and independent from the industry evidence. The extent to which TTCs disclosed financial conflicts of interest (COI) when citing evidence was examined.

**Results:**

We obtained 74 pieces of TTC-cited evidence. The quality of the evidence was poor. TTCs cited no independent, peer-reviewed evidence that supported their arguments. Nearly half of the evidence was industry-connected (47%, 35/74). None of this industry-connected evidence was published in peer-reviewed journals (0/35) and 66% (23/35) of it was opinion only. Industry-connected evidence was of significantly poorer quality than independent evidence (p<0.001). COIs were not disclosed by TTCs in 91% (32/35) of cases.

**Conclusions:**

In the absence of peer-reviewed research to support their arguments, TTCs relied on evidence they commissioned and the opinions of TTC-connected third-parties. Such connections were not disclosed by TTCs when citing this evidence and were time consuming to uncover. In line with Article 5.3 of the Framework Convention on Tobacco Control and broader transparency initiatives, TTCs should be required to disclose their funding of all third-parties and any COIs when citing evidence.

## Introduction

Transnational tobacco companies (TTCs) have vehemently opposed proposals for the introduction of standardised packaging (SP) for tobacco products in the UK,^1–9^ as they have in other countries where governments have attempted to introduce this policy.^10–15^ In submissions to the Department of Health's 2012 consultation on this issue, TTCs argued the policy would have unintended negative economic consequences, including a detrimental impact on retailers, manufacturers and the exchequer, and an increase in the illicit tobacco trade.^1–4^ ^8^ ^16^ Leaked Philip Morris International (PMI) documents, which outlined the company's plans to prevent SP in the UK, confirm that the company planned widespread dissemination of these arguments to the public and to decision makers to promote their main message that the government's ‘focus needs to be on economy and growth’.^17^ ^18^

Such economic arguments can be powerfully used under the framework of Better Regulation (BR), introduced from the mid-1990s across jurisdictions globally, including the EU and UK. Better, or smart, regulation aims to improve policy-making by increasing the role of evidence in policy-making as well as formalising opportunities for affected interests to be consulted.^19^ However, BR has increasingly become a system for reducing regulation and enhancing business competitiveness.^19^ ^20^ In the UK, government guidelines on BR now make it explicit that regulation should not ‘impose costs’ on business ‘unless a …compelling case has been made’.^21^ Impact Assessment and stakeholder consultation, the tools through which potential costs and benefits of proposed regulation^22^ are assessed, give corporations an explicit role in assessing and contesting the evidence for a policy.^23^ Previous research suggests that the use of a cost benefit approach to impact assessment used within this system confers advantages on well-resourced companies seeking to oppose regulations that threaten their profits.^24^ British American Tobacco (BAT) played an instrumental role in embedding BR tools into EU policy-making, anticipating that they would make it harder to pass public health policies.^25^

In light of the above, TTCs’ long-standing history of manipulating evidence in their own interest^26–33^ is of particular concern. We have already shown that the evidence used by TTCs in their submissions to argue that SP will not reduce smoking uptake was of low quality,^8^ and TTCs repeatedly misquoted and distorted the main messages of published studies.^9^ TTCs’ misuse of illicit trade and economic arguments has been well documented.^34^ ^35^ Their claims that SP would increase the illicit trade in tobacco in Australia and lengthen transaction times, because generic packets would take longer for sales staff to locate, have been roundly refuted by emerging evidence.^36–39^

The situation is further complicated by TTCs’ growing use of the third-party technique: using a seemingly independent messenger with a better reputation and greater credibility to convey arguments.^40^ Previous efforts by TTCs have included hiring independent organisations and experts, creating front groups and establishing alliances with lobby groups and other industries to campaign on their behalf.^25^ ^41–43^ The aforementioned PMI leaked documents also reveal that third-parties were to play a central role in disseminating its economic and illicit trade arguments against SP.^17^ ^18^

This paper therefore aims to assess the quality and relevance of the evidence that TTCs cited in their submissions to the UK's 2012 consultation on SP to argue that the policy would lead to negative economic impacts and an increase in the illicit tobacco trade. It also examines the degree to which TTCs cite ‘evidence’ from those with TTC connections (ie, third-parties) and how transparent TTCs are about these connections in their written submissions. The UK Intellectual Property Office's guidance on standards of evidence used in the development of policy state: “An important part of public policy-making is transparency. It should be made clear who has commissioned and funded the research as well as who has carried it out.”^44^ This is compatible with principle 3 of Article 5.3 of the Framework Convention on Tobacco Control: “Parties should require the tobacco industry and those working to further its interests to operate and act in a manner that is accountable and transparent.”^45^

## Methods

### Data

Consultation submissions were obtained, via company websites, for the four TTCs operating in the UK—Imperial Tobacco, Japan Tobacco International (JTI), BAT and PMI. Every citation (hereafter referred to as evidence) made in each TTC submission to support arguments on alleged illicit trade and economic impacts of SP was recorded. In line with questions asked in the Department of Health's Impact Assessment,^46^ we defined economic evidence as that which discussed: production and distribution costs to manufacturers, costs to retailers and the public relating to transaction times, or costs to the exchequer through loss of tobacco duty. Arguments that concerned economics but focused on individual behavioural impacts, for example, that SP will lead to increased price competition and therefore increased consumption, were excluded. Illicit trade evidence was any citation used to argue that SP would impact on the illicit tobacco trade.

Unlike our previous work, where we recorded only formal written evidence cited,^8^ the scant use of formal evidence and substantially greater use of informal evidence such as press coverage and individual commentaries on economic/illicit issues meant that we interpreted evidence broadly. We included all economic/illicit ‘evidence’ referred to in the text and cited in the bibliography of each TTC's submission in addition to quotes in the main text that were attributed to individuals or organisations but not formally cited. Where more than one company had cited identical evidence it was only counted once.

### Analysis

Copies of evidence cited by TTCs were obtained and for each, the author, title, date and source were recorded in an Excel spreadsheet. The quality of the evidence and its relevance to SP was assessed using criteria based on existing literature in this field.^8^ ^27^ ^47–51^ Additional categories were developed to allow for the generally low quality of the evidence (table 1).

**Table 1:** Coding framework for classifying evidence

	Evidential criteria	Basis in existing literature	Data coding framework	Coding categories
Quality	Independence	Who funded the evidence? Are authors affiliated to the tobacco industry?^27^ ^47^ ^49^ ^51^	Who funded the research? Has the author of the research any connection with the tobacco industry?	▸ Tobacco industry-connected (author TTC employed, TTC created, TTC commissioned, TTC part-funded, part of TTC supply chain, received TTC hospitality) ▸ Independent of the tobacco industry ▸ Unknown (could not be determined either way)
	Nature of the evidence	Is the evidence a research study or is it something else?	What was the evidence composed of? Was it a piece of research? If not what was it?	▸ Research (primary research carried out by the author, or secondary research evaluating/summarising two or more primary research studies) ▸ Publication of facts and/or figures only (no opinion expressed) ▸ Strategy document (outlines a strategy or plan of action, eg, organisation annual report including evaluation of previous year and plans for the future) ▸ Opinion with or without supporting evidence (referenced evidence, data, figures, casual references to the evidence with no formal citation and opinion with no supporting evidence at all)
	Publication Route	Has the evidence been peer-reviewed or published via traditional academic routes?^27^ ^47^ ^49^ ^50^	Was the research published in a peer-reviewed journal or another legitimate research avenue? If not, where was the research published?	▸ Academic (peer-reviewed journal articles, other academic including conference papers, research reports, evaluation reports) ▸ Official government (eg, government report, policy document, commissioned review, speech, statement, website, opinion, briefing, newsletter, summary, press release) ▸ Official parliamentary Publication (eg, House of Commons questions in Hansard) ▸ Private publication by company/organisation (can include report, consultation response, briefing, summary, newsletter, factsheet, webpage content, press release, private letters, blog) ▸ Press article or media coverage (newspaper, trade magazine, published letters, tv programme)
Relevance	Subject matter	What is the topic, argument, position or conclusion of the evidence?^27^ ^49^ ^50^	What issue does the research address?	Either illicit trade and/or economic issues, and: ▸ SP/tobacco packaging (‘Highly relevant’) ▸ Tobacco not packaging or unrelated to either packaging or tobacco (‘Less relevant’)

Table amended from Hatchard *et al*.^8^

SP, standardised packaging; TTC, transnational tobacco company.

#### Quality

Evidential quality was assessed using three criteria: independence from the tobacco industry, nature of the evidence and publication route (table 1).

**Figure 1:**
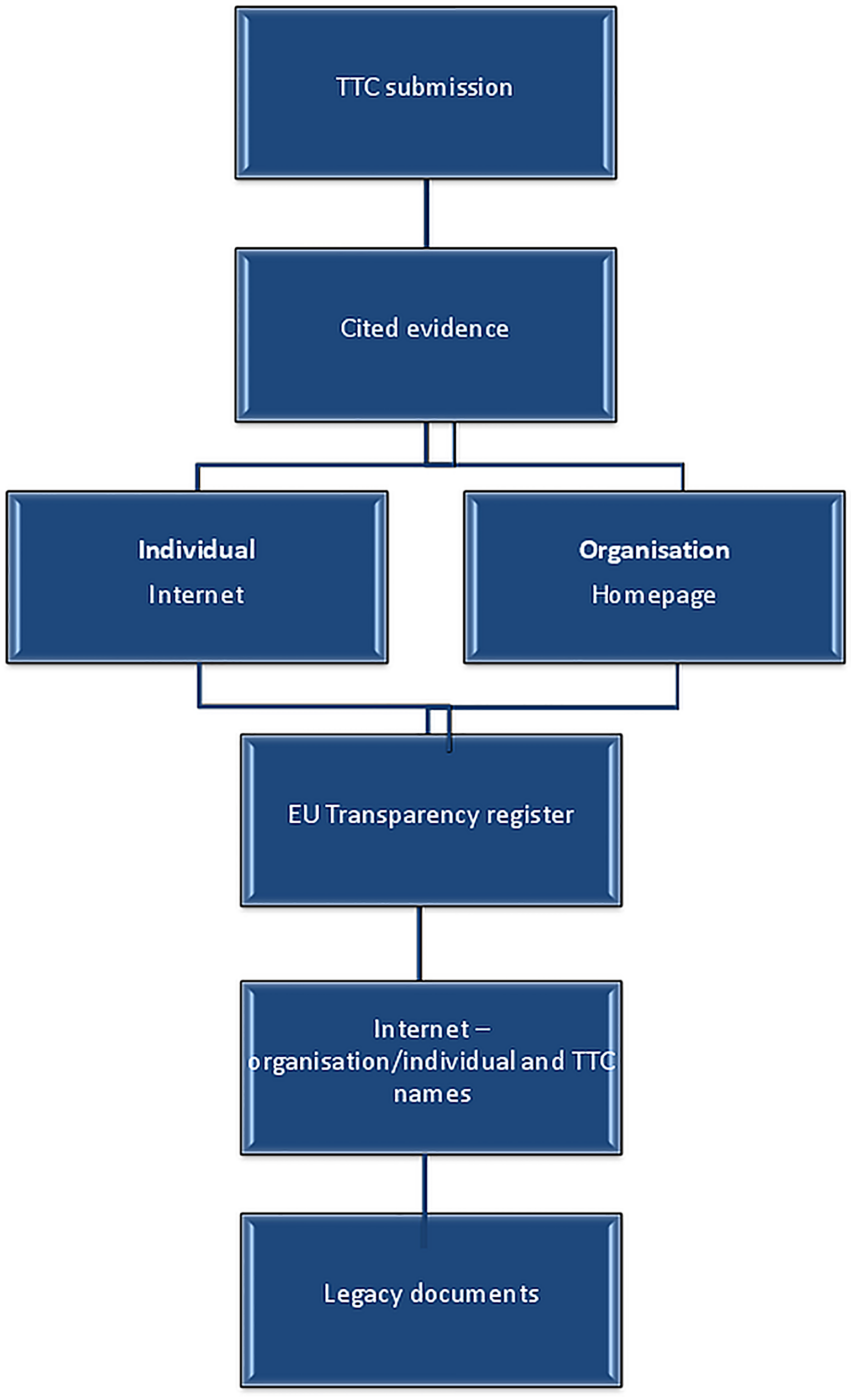
Procedure followed when searching for any conflict of interest between transnational tobacco companies (TTCs) and the individuals and organisations cited. *Note*: Could have changed the order of this procedure by searching immediately for organisations or individuals alongside the names of TTCs, or searching the legacy library. However, we were interested in how transparent TTCs, individuals and organisations were, and this provided justification for the order in which these searches were conducted.

*Independence*: For each piece of evidence cited, the TTC's submission was searched for a funding or a conflict of interest (COI) statement. If no disclosure was found, we noted that the TTC had not declared any financial relationship between themselves and the authors of the cited evidence. At this stage, to clarify whether the evidence was either connected or independent of industry, each piece of evidence was searched for a funding or COI statement. If none was present within the evidence document itself then a further series of checks was performed (see figure 1). Evidence was categorised as independent of the tobacco industry if it included a clear funding statement that did not mention tobacco industry funding or if further internet searches revealed no relationship existed between the author and any tobacco company. Evidence was categorised as industry-connected if there was evidence of a financial relationship between its author (whether individual or organisational) and one or more TTC. The degree of financial relationship varied on a continuum from complete to part funding (table 1).

As space precludes us from providing full details of the industry-connected organisations and individuals identified during the course of this research, these are available on our website, http://www.TobaccoTactics.org.

The *nature of the evidence* was categorised as either research (eg, interviews, surveys, observational studies, experimental studies, systematic reviews, literature reviews and critiques), statistics or facts with no opinion expressed (eg, presentation of data without the underlying methodological detail found in research studies), opinion (including those with some supporting evidence ranging from referenced evidence, data, figures, to casual references to ‘the evidence’ and those that made no attempt to refer to any evidence in either a formal or casual capacity), or strategy documents (primarily outlining a plan of action, including a combination of research, opinions, and/or facts and figures; table 1).

*Publication route* was categorised to account for the diversity of evidence. Although peer-reviewed studies are considered to be superior quality evidence,^52^ many other forms are also submitted to, and considered in, public consultations.^53–57^ Our categorisation included peer-reviewed journals and other academic outlets, official government publications, official parliamentary publications, publication by private companies and organisations, and those published by the press (table 1).

#### Relevance

To determine its relevance to SP policy, the *subject matter* of each piece of evidence was recorded. Evidence was coded as ‘highly relevant’ if it was about illicit trade or economic impacts (see above) *and* tobacco packaging, standardised or otherwise. Evidence about illicit trade or economic issues but *not* packaging was coded as ‘less relevant’ to SP regulation.^8^

Evidence was coded by two researchers (KAER and JH) with 94% concordance. All discrepancies were resolved.

Following categorisation of the evidence, we compared the relevance and quality of the evidence connected to and independent of industry using two-tailed Fisher's exact tests. The measures of quality used for this analysis were the nature of the evidence and publication route. For nature of the evidence we combined research, publication of facts and statistics, and strategy documents and compared these with opinion. For publication route, we combined articles published in peer-reviewed journals or other academic outlets along with official government and parliamentary publications and compared them with those published by private organisations or in the press.

Finally, for TTC-cited evidence coded as industry-connected, the number of instances where TTCs had disclosed this link in their submissions was recorded.

## Results

Imperial, JTI, BAT and PMI collectively cited 83 pieces of unique evidence to support illicit trade and economic arguments against SP. We were able to obtain 74; 11 were predominantly about economic issues, 50 were about illicit issues, while 13 pieces of evidence referred to both. Examples of these arguments and the evidence used to support them are given in box 1.

Box 1 **Transnational tobacco companies (TTCs) use of evidence to support economic and illicit arguments against standardised packaging**

**Negative economic consequences**

“The Tobacco Retailers Alliance UK, a coalition of 26 000 independent shopkeepers who sell tobacco products, has warned its members that: ‘…banning cigarette branding would directly threaten small shops. For many of you, tobacco sales make up around a third of turnover, sometimes more. . . . During the busy times, such as the morning rush, there is a real risk that customers who have to wait will go to supermarkets and larger shops, which have more staff and therefore shorter transaction times.’”[British American Tobacco,^4^ 2012 citing evidence from the Tobacco Retailers Alliance]^61^

In its submission BAT does not disclose that the Tobacco Retailers Alliance is funded by the Tobacco Manufacturers’ Association, which in turn is entirely funded by three TTCs, including BAT.^62^ ^63^

Two reports prepared by Deloitte for the Alliance of Australian Retailers^72^ ^73^ suggest that retailing times would be adversely affected if standardised packaging were introduced by an additional 15 to 45 seconds per transaction and that the effect would be particularly significant for smaller retailers. [Imperial Tobacco, 2012]^3^

In its submission, Imperial Tobacco does not disclose that the Alliance of Australian Retailers is a front group created and funded by PMI, Imperial and BAT and managed daily by PMI personnel.^135^

**Independent evidence finds that illicit tobacco trade will worsen**

“The expert opinion of Professors Zimmerman and Chaudhry is that plain packaging ‘will worsen the illicit trade in tobacco products’. JTI shares this view.” [Japan Tobacco International, 2012]^2^

In its submission JTI does not disclose that it commissioned the Chaudhry and Zimmerman report.^71^

“Experts from law enforcement officers to academics have concluded that plain packaging will increase demand for illicit tobacco products. For example, plain packaging will likely cause an increase in the black market for smuggled branded tobacco according to nearly 70% of current UK police officers who responded to a recent survey” [Philip Morris, 2012]^1^

In its submission PMI does not disclose that it commissioned the police officers survey^76^ or that the academics they cite therein have been commissioned to produce reports. Nor do they disclose that the two ex-law enforcement officers that they cite, Roy Ramm^114^ and Peter Sheridan,^115^ are co-founders of the Common Sense Alliance, which is funded, at least in part, by British American Tobacco.^136^

### Quality

Overall, the quality of the cited evidence was very poor; this was particularly the case for industry-connected evidence (table 2).

**Table 2:** Number of TTC-cited pieces of evidence by relevance and quality (nature, publication route) and independence from the tobacco industry (n=74)

	Relevance		Nature of the evidence			Publication route					
Quality indicators Connection with industry	Packaging	Other	Research	Opinion	Other	Peer-review	Academic other	Official government/ parliament	Private	Press	Total
Independent	9	29	10	9	19	2	2	24	6	4	38
Connected	32	3	9	23	3	0	1	0	27	7	35
Unknown	1	–	–	1	–	–	–	–	1	–	1
Total	42	32	19	33	22	2	3	24	34	11	74

TTC, transnational tobacco company.

### Independence

Almost half of the evidence cited (35/74, 48%) was financially connected with the tobacco industry, while 51% (38/74) was independent of the tobacco industry. For one piece of evidence it could not be determined either way.

#### Nature of evidence

**Figure 2:**
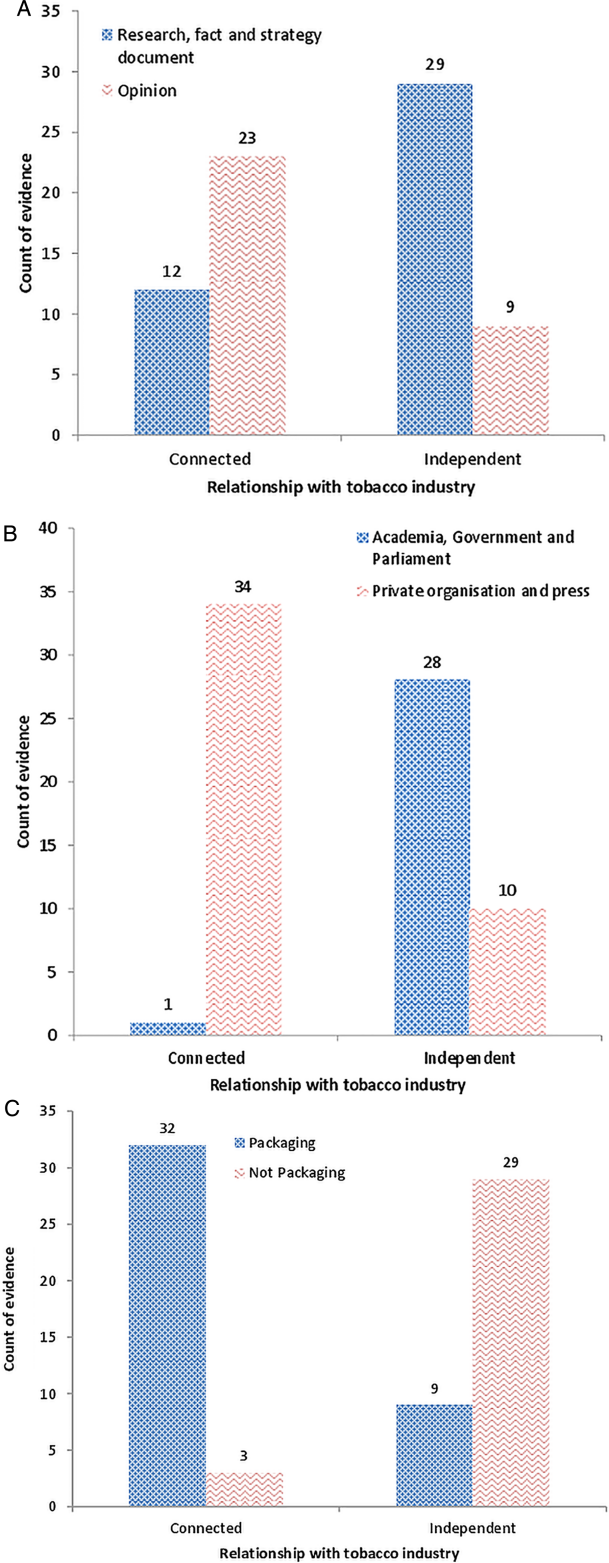
(A) Nature of the evidence by tobacco industry connection (N=73*). (B) Publication route by tobacco industry connection (N=73*). (C) Relevance of evidence by tobacco industry connection (N=73*). *Note: N=73 because one piece of evidence (relevant to private organisation and press) could not be classified as either connected or independent of the tobacco industry.

Nearly half of the evidence consisted of opinion only (45%; 33/74; table 2). A quarter (19/74) was research, 15% (11/74) were strategy documents and a further 15% were facts and figures. The majority of industry-connected evidence was opinion (23/35, 66%), while the majority of the independent evidence 76% (29/38) comprised research, facts and figures or strategy documents (figure 2A). These differences in quality were highly significant (p<0.001).

#### Publication route

Only 7% (5/74) of the evidence was published in either a peer-reviewed journal (2) or other academic outlet (3), while 46% (34/74) was privately published, 32% (24/74) came from official government or parliamentary sources and 15% (11/74) was published in the press (table 2). The publication route of industry-connected and independent evidence differed significantly (p<0.001). None of the industry evidence was peer-reviewed, the majority, 97% (34/35), instead being published privately (27/35) or in press articles (7/35; figure 2B). In contrast, 74% (28/38) of evidence independent of the tobacco industry was research, government or parliamentary publication.

#### Relevance

Of the 74 pieces of evidence obtained 42/74 (57%) were relevant to either SP or packaging in general, 31/74 (42%) about tobacco but not packaging and 1/74 (1%) was unrelated to either packaging or tobacco. The odds of evidence being about packaging were 34 times higher if it was industry-connected (32/35) compared to independent evidence (9/38; p<0.001; figure 2C). None of the independent, peer-reviewed evidence that TTCs cited were highly relevant to SP (0/2).

### Transparency around industry-connected evidence

In only 3 of 35 instances did TTCs declare their financial links to the organisations and individuals authoring the evidence they cited. In two of these instances this could not be avoided as JTI was actually the author.

**Figure 3:**
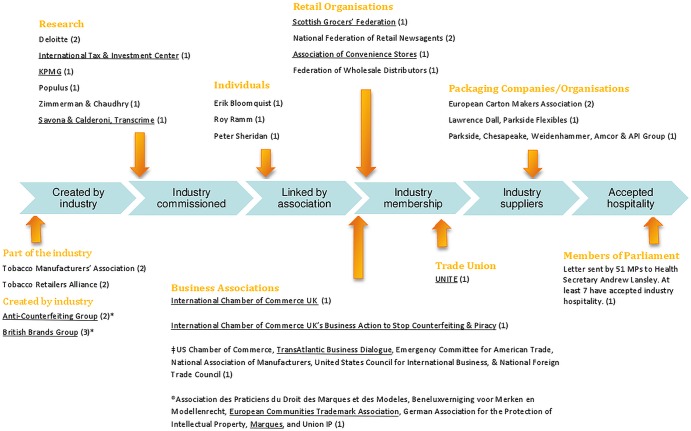
Connection to Transnational Tobacco Companies (TTCs, N=32 pieces of evidence). *Note*: Numbers in parentheses refer to the number of unique references authored by the named organisation(s) or individual(s). Those underlined were identified by Philip Morris International as either ‘influencers’ or ‘messengers’.^17^ ^18^^†^In the joint statement cited by these business associations, we were unable to determine whether the following business associations have TTC members: the Emergency Committee for American Trade. However, links were found between the remaining five authors. ^®^4/6 of the business associations authoring this joint statement were industry-connected, however, we were unable to determine the existence of a link between Association des praticiens du droit des marques et des modèles (APRAM) or Union IP (an intellectual property organisation). *The Anti-Counterfeiting Group and British Brands Group are joint authors of two pieces of unique evidence.

TTCs did not disclose their links to the authors of the remaining 32/35 (91%) pieces of evidence. The authors of this linked evidence were either TTC employed, created, commissioned, part-funded (eg, by membership fees), involved in the tobacco supply chain or in receipt of TTC hospitality (figure 3).

Only in two instances (two pieces of evidence authored by the *Tobacco Manufacturers’ Association—*the TTCs’ trade association in the UK^58^ ^59^) might readers be expected to identify the industry links. In all other instances, links to tobacco companies would have been difficult to identify. For example, TTCs cited two pieces of evidence from the *Tobacco Retailers Alliance*,^60^ ^61^ without disclosing that it is a front group *created* and entirely financed by the Tobacco Manufacturers’ Association.^62^ ^63^ A further four pieces of cited evidence were authored by two organisations, the *British Brands Group* and the *Anti-Counterfeiting Group*,^64–67^ both of which were originally *created* by one or more TTCs.^7^ ^68–70^ Although all four TTCs cited evidence from the *British Brands Group*, neither Imperial Tobacco, BAT nor PMI disclosed that they were members. However, it must be noted that JTI, which is not a member, did disclose in its submission that although it was not a member, other tobacco companies were.

TTCs cited seven research reports they had *commissioned* (see figure 3 for authors), but did not declare this fact in their submissions (a funding statement was included in each of the reports but this would require policymakers to access these reports directly in addition to reading each TTC submission).^71–77^ A further nine pieces of cited evidence were attributed to organisations in receipt of tobacco company *funding* (eg, with one or more fee-paying TTC members), which was not declared.^78–92^ This evidence included four opinion statements, two from two sets of business associations^93^ ^94^ one from the *International Chamber of Commerce* (ICC)^95^ and one from its subsidiary, the *ICC Business Action to Stop Counterfeiting and Piracy*.^96^ Three press articles and one response to the Department of Health's 2008 Consultation on the Future of Tobacco Control cited the opinions of four different retail organisations,^97–100^ and a press release stating the opinion of Trade Union *UNITE*, 101 whose members include TTC employees.^102^ TTCs did not disclose any links with these organisations when it cited this evidence.

Four pieces of evidence were authored by *supply chain* companies, two from packaging companies (who list TTCs as clients)^103^ ^104^ and two from the *European Carton Makers Association*,^105^ ^106^ an organisation representing the interests of packaging companies, including those making tobacco packaging.^107^ ^108^ These links were not declared by tobacco companies in their submissions.

An open letter cosigned by 51 Members of Parliament to Health Secretary Andrew Lansley^109^ included seven signatories who had accepted *hospitality* from JTI.^110^ This link was also not declared by the citing tobacco company.

Finally, in three cases, the authors’ links to the tobacco industry were more obscure. A report by *Erik Bloomquist*, an equity analyst at Berenberg Bank, was cited.^111^ This report credited tobacco industry consultant John Luik^112^ for his assistance. Plagiarism software, WCopyfind V.4.1.1, revealed that 73% of a report called ‘Erasing intellectual property’, authored by Luik and Patrick Basham,^113^ is replicated in Bloomquist's report.^111^ Given the known relationship between Patrick Basham, John Luik and TTCs,^112^ an indirect link between Bloomquist's report and the tobacco industry was assumed. Similarly the opinions of two former senior police officers, Roy Ramm and Peter Sheridan, were cited.^114^ ^115^ However, it was not disclosed that both are cofounders of the *Common Sense Alliance,* an antiregulatory lobby group that receives financial support from BAT.^136^

## Discussion

### Key findings

The evidence TTCs used to support their argument that SP would have adverse economic and illicit trade impacts was of very low quality. TTCs did not cite any independent, peer-reviewed evidence that supported these arguments. Instead they relied on research they had commissioned^69–75^ or the opinions of those with varying degrees of financial links to the industry. Of the 35 industry-connected pieces of evidence, seven were research reports commissioned by TTCs or by a third-party financially connected to a TTC, and the majority of the remainder (23/35) were opinions of business and retail organisations with TTC members, packaging companies with TTC clients, a trade union with members who are TTC employees and MPs sympathetic to the industry position on SP, some of whom have taken TTC hospitality. Although nearly half of the evidence cited by TTCs was financially connected to them, the companies did not disclose this connection in 91% of cases and such connections were often time-consuming to discover.

### Limitations

We set the parameters of this study to focus exclusively on the evidence TTCs used to support illicit trade and economic arguments (as defined in the Impact Assessment)^46^ and have not, therefore, examined all the evidence cited in their submissions to support all their arguments. Nor do we assess the accuracy of the TTCs’ interpretation of the evidence cited. However, we have previously examined the quality and relevance of the evidence TTCs used to argue that SP will not reduce youth smoking uptake,^8^ and undertaken in depth analysis of how they interpreted evidence in their submissions, identifying a number of techniques used to misrepresent and distort evidence.^9^

As we were interested specifically in the quality of the evidence used by TTCs, we only counted unique pieces of evidence, although many were cited by more than one company and sometimes by all four. Finally, we were unable to identify industry links to three of the business organisations who co-authored, with other TTC-connected business organisations, opinion statements cited by the TTCs (ECAT, APRAM, UNION-IP; figure 3). The policy of private membership implemented by many business organisations made it difficult to determine any links. However, due to the financial links of the coauthors of these pieces of evidence, the individual pieces of evidence were coded as industry-connected.

### Discussion/links with previous evidence

Our findings support previous research, which shows that, in response to regulatory proposals, evidence submitted by the tobacco industry has typically been less scientifically rigorous than the evidence cited by supporters of the regulation.^115^ They also add to a growing body of literature, which shows that, in order to oppose SP, TTCs cited poor quality, industry-funded evidence that purported to show the policy would not work^8^ and fundamentally misrepresented independent evidence supportive of the policy.^9^

In relation to illicit trade, our findings provide further evidence that TTC-commissioned data and evidence on illicit trade cannot be trusted. TTC data across a number of jurisdictions, including that commissioned from leading accountancy firms, have been shown to over-estimate the scale of the illicit trade, exaggerate the upward trend (including by revising historical figures downwards) or misrepresent the nature of the trade in order to down-play the extent of tobacco industry involvement.^5^ ^118–123^ The limited methodological detail available on the tobacco industry's empty pack surveys, which usually form the basis of these industry data, suggest they may be deliberately designed to exaggerate the extent of the illicit trade.^121^ In a retreat following criticism of its previous data,^121^ KPMG's latest report for PMI has revised its illicit estimate for the UK illicit trade downwards stating “alternative data sources suggest this [the 2012 estimate] may have overstated non-domestic incidence for the full year”.^124^ Industry claims that various policies, including SP, will fuel the illicit trade have been found to be highly misleading.^34^ ^35^ ^125^ For example, following the introduction of SP in Australia, another KPMG report produced on behalf of PMI claimed there had been a dramatic increase in the illicit tobacco trade in the country.^126^ However, in addition to criticisms of its methodology,^118^ independent research found no evidence of increased illicit use.^39^ ^127^

Taken together, the absence of independent evidence, or even independent opinion, that SP will increase illicit trade revealed in this paper, the questionable veracity of industry-funded data, the fact that the industry's previous illicit trade predictions have not materialised and PMI's explicit intention to utilise the illicit trade argument to “ensure that PP [plain packaging] is not adopted in the UK”,^17^ suggest that the illicit trade argument is being used as an industry tactic to discourage governments from pursuing further tobacco control policies.

The same is true of TTCs’ claims that SP will have adverse economic consequences. Independent research has found no evidence to support industry predictions that small businesses in Australia would suffer from customers moving from small convenience stores to bigger supermarkets.^39^

TTCs extensive use of third-party voices as authors of the ‘evidence’ creates problems for policymakers trying to assess the reliability of evidence. Tobacco companies’ failure to declare these COI in their 2012 consultation submissions demonstrates that, despite decades of criticism and a requirement for transparency in Article 5.3, TTCs continue to rely heavily on the third-party technique to disseminate their messages via those with more credible voices than their own. Furthermore, the number and nature of organisations and individuals cited creates the impression of a large, expert and seemingly unconnected cohort, all opposed to or concerned about SP. PMI's leaked SP-opposition strategy named many (46%, 12/26 listed figure 3) of the individuals and organisations cited in the TTC submissions (in the context covered in this paper) as ‘influencers’ or ‘media messengers’ (figure 3).^17^ By naming these influencers and messengers and giving a timeline indicating when third-parties would issue press releases and reports, the documents imply that PMI may have had some degree of influence.

### Policy implications

Our findings provide further evidence that BR is problematic for the development of tobacco control policy. Within this regulatory environment, stakeholders (including corporations) are granted an explicit role to provide new evidence and to contest existing evidence and data. However, inadequate COI disclosure requirements mean that BR fails to take account of TTCs’ longstanding history of manipulating evidence to serve their own interests. In this instance, the BR framework has enabled TTCs to more effectively frame and use their misleading arguments. This has contributed to uncertainty about policy impacts and consequent regulatory delays.^128–130^ PMI recognised this potential in BR as is revealed in their leaked documents,^17^ while BAT predicted, in pushing for BR, that it would enable them to contest, delay and ultimately overturn policies.^25^ The BR agenda, particularly the requirement for stakeholder consultation, can be seen to directly undermine the purpose of Article 5.3—to protect public health policies relating to tobacco control “from commercial or vested interests of the tobacco industry”^45^—and to legitimise TTCs’ counter-argument that they ought to be involved in decisions that affect them.^25^ ^131^

Given the findings of this paper, the requirements of Article 5.3 and the overwhelming evidence of the tobacco industry's misuse of evidence, the terms of their inclusion in stakeholder consultation processes require some reassessment. *If* TTCs are allowed to continue to submit evidence to public consultations as part of BR, it should be compulsory for them, in line with 5.3, to exercise transparency by disclosing any potential COI, not only of their own position, but also that of the evidence they use to build their case either for or against a policy proposal. Such disclosures are now standard practice when submitting research to health-related academic journals.^132–134^ Where TTCs are found to have omitted such disclosures, governments should not be required to consider this evidence in their policy deliberations. Further, any data produced by industry on the illicit tobacco trade, or on those with whom it has a financial relationship, should be treated with extreme caution.

**What this study adds**

- Our analysis reveals that transnational tobacco companies (TTCs) have cited no independent, peer-reviewed evidence that supports their case. Instead they relied on evidence they had commissioned and the opinions of TTC-connected third-parties, providing further evidence that TTCs exaggerate the threat of illicit tobacco and the negative economic consequences of policy as part of a deliberate strategy to ward off regulation.
- TTCs have not disclosed relevant conflicts of interest within their submissions, highlighting incompatibility between Better Regulation and Article 5.3 of the Framework Convention on Tobacco Control.
